# Strengthening pharmacovigilance and regulatory capacity in Southern Africa (SPaRCS): achievements, experiences and lessons learned from a participatory action learning project

**DOI:** 10.3389/fdsfr.2026.1765077

**Published:** 2026-05-29

**Authors:** Michelle Viljoen, Star Khoza, Mukesh Dheda, Libert Chirinda, Frieda Shigwedha, Anna Shimbulu, SipheSihle Nhlabatsi, Nomsa Shongwe, Albert Figueras, Raffaella Ravinetto, Hazel Bradley

**Affiliations:** 1 School of Pharmacy, University of the Western Cape, Cape Town, South Africa; 2 Pharmacovigilance Centre for Public Health Programs, National Department of Health, Pretoria, South Africa; 3 Pharmacovigilance and Clinical Trials Division, Medicines Control Authority of Zimbabwe, Harare, Zimbabwe; 4 Namibia Medicines Regulatory Council, Ministry of Health and Social Services, Windhoek, Namibia; 5 Pharmaceutical Services, Ministry of Health, Mbabane, Eswatini; 6 Independent Consultant, Barcelona, Spain; 7 Department of Public Health, Institute of Tropical Medicine, Antwerp, Belgium; 8 School of Public Health, University of the Western Cape, Cape Town, South Africa

**Keywords:** capacity building, clinical trials, collaborative partnership, community health workers, pharmacovigilance, regulation, safety, Southern Africa

## Abstract

Effective regulatory systems play a critical role in improving access to safe, efficacious and quality medicines and vaccines. However, most regulatory and pharmacovigilance (PV) systems in sub-Saharan Africa (SSA) are still under-resourced and lack recognition of their public health service role, despite encouraging developments in several countries, and positive experiences of harmonization across the continent. The aim of Strengthening pharmacovigilance and regulatory capacities in four Southern African countries (SPaRCS), was to improve pharmacovigilance and clinical trials oversight in Namibia, Eswatini, Zimbabwe and South Africa. The project used a participatory action learning and co-creation approach and built on existing relationships with national regulatory authorities and PV centres in the four countries. Key activities included a mapping of PV and clinical trials oversight in the four partner countries; participatory workshops and learning exchanges in two thematic areas; Strengthening PV systems and Clinical trials oversight, and collaborative development of a training package for community health workers on adverse drug reaction reporting. Intentionally SPaRCS provided opportunities for partners in the four countries to develop relationships amongst those involved in PV and clinical trials oversight in the region. This included sharing of challenges, field experiences and best practices in a sub-national, national and regional setting. Strong commitment of country partners and the flexible learning and co-creation approach facilitated positive achievements. Strengthening PV and clinical trial oversight requires a multi-faceted approach involving the establishment of robust systems, addressing infrastructure and training gaps, integrating PV into public health programs and building formal collaborative platforms at regional level. The regional platform created by SPaRCS provided a starting point, for the establishment of the Centre of Excellence for Pharmacovigilance in Southern Africa (CEPSA) launched at the end of 2024.

## 1 Introduction

Access to safe, effective and quality-assured medicines and vaccines is one of the important goals of the United Nations Sustainable Development Goals (SDG3.8). (United Nations General Assembly, 2015). Effective regulatory systems for health products are recognized as essential components of strengthening health systems as they play a critical role in improving access to health products, through a complex set of interconnected systems including, but not limited to market authorization, licensing of pharmaceutical premises, and continuous monitoring of the safety, efficacy and quality of medicines through market surveillance and pharmacovigilance. (World Health Organization, 2021). The COVID-19 pandemic highlighted the pre-existing disparities in access to health products, particularly between the Global North and Global South and across sub-Saharan Africa (SSA), and demonstrated the need for a global rethinking of the pharmaceutical ecosystems, including supporting pharmaceutical Research & Development (R&D) and production within the continent. (Ravinetto et al., 2024). This renewed focus on pharmaceutical R&D and manufacturing in SSA acted as a powerful reminder of the need for stringent regulatory control in terms of clinical trials oversight, marketing authorization and post-marketing surveillance, including pharmacovigilance and quality monitoring in both clinical and community settings.

The crucial role of pharmacovigilance is often underestimated in policy-making. Pharmacovigilance (PV) is defined by the World Health Organization (WHO) as the “science and activities relating to the detection, assessment, understanding and prevention of adverse effects or any other medicine-related problem”. (World Health Organization, 2004). The COVID-19 vaccination programs highlighted, in particular, the importance of having well-prepared PV systems in place globally. (Naniche et al., 2021). Yet, most regulatory and PV systems in SSA are still under-resourced in terms of human and financial capacity. In low and middle-income countries (LMICs), underreporting and inadequate use of data are the main issues that traditionally hinder the full potential of PV systems. (Kiguba et al., 2023). Moreover, policymakers in these countries frequently do not recognize the public health role of pharmacovigilance.

Regulatory strengthening initiatives traditionally prioritized the pre-market activities, e.g. the capacity to issue licenses and marketing authorization, while less focus was put on surveillance. The emphasis on pre-marketing activities can be explained by the imperative to demonstrate that medicines and vaccines are safe and not toxic (Newton et al., 2019; Ozawa et al., 2020; World Health Organization, 2017). These pre-marketing activities include clinical trials oversight playing an important role in ensuring the safety, efficacy, scientific and ethical integrity of research which is critical for maintaining public trust. However, post-market surveillance and particularly PV are essential to protect public health and to enhance the performance of health systems, by *detecting* and *responding to* safety and quality issues once a medicine or vaccine is on the market. (Toroitich et al., 2024). This is because before being put on the market, a new medicine or vaccine has only been tested in a relatively small sample of patients under controlled conditions, so there is insufficient knowledge of the effects of these products when used in real-world conditions. (Onakpoya et al., 2016; Wang et al., 2023).

There are some encouraging recent developments. For example, several African countries have achieved maturity level 3 (ML3) for medicines or vaccines, based on the WHO Global Benchmarking Tool for regulatory authorities. ML3 indicates a stable, well-functioning, and integrated regulatory system for monitoring the safety of health products. Ethiopia Ghana, Nigeria, Rwanda, Senegal, Tanzania and Zimbabwe are already functioning at ML3 for medicines and vaccines (vaccines non-producing), Egypt at ML3 for medicines and vaccines (producing) and South Africa at ML3 for vaccines (producing). (World Health Organization, 2024). Furthermore, there are positive experiences from regional harmonization initiatives from the East African Community (EAC); (Mashingia et al., 2023); the Southern African Development Community (SADC) (Kniazkov et al., 2020) and ZaZiBoNa; (Dube-Mwedzi and Suleman, 2025); the Economic Community of West African States (ECOWAS), (Mace et al., 2023), and the Intergovernmental Authority on Development. (Intergovernmental Authority for Development IGAD, 2025). For example, in the EAC these initiatives include conducting joint assessments and developing a Post-Marketing Surveillance Strategy. (Mashingia et al., 2023). The recently founded African Medicine Agency (AMA), modeled on the European Medicines Agency, could be a game changer in further enhancing regulatory harmonization, supporting both pharmaceutical production and safety monitoring of health products at continental level. (Mace et al., 2023; Duga et al., 2025; Ndomondo-Sigonda et al., 2023; Ogbodum et al., 2023).

To institute a strong, cohesive and sustainable PV System, embedded in and linked to a stringent medicine regulatory environment, requires resource-intensive commitments in funding, infrastructure, human capacity, expertise, and cross-country collaboration, as well as strong political will to commit to these endeavors. (Amp et al., 2018; Mehta et al., 2017; Ndagije et al., 2023). This case study describes the achievements, experiences and lessons learned from the three and a half year project *Strengthening Pharmacovigilance and Regulatory Capacities in four Southern African countries (SPaRCS)*, (SPaRCS, 2020), which commenced in April 2020 with the aim of strengthening PV systems and clinical trials oversight of national regulatory authorities (NRAs) in the Namibia, Eswatini, Zimbabwe and South Africa to develop personal and institutional capacities.

## 2 Context

SPaRCS was led by South Africa’s University of the Western Cape (UWC), School of Public Health and School of Pharmacy, in collaboration with NRAs from Namibia, Eswatini and Zimbabwe and the South African National Department of Health. The initial idea for the project was conceived by the UWC team who invited partners from the four countries, with whom they had existing relationships, to collaborate and develop the project proposal together. Three objectives were identified: 1. To understand the regulatory systems as they relate to PV and clinical trials oversight in the four Southern African countries, and to identify actors, processes and communication pathways, good practices, gaps and opportunities for collaborative learning; 2. To facilitate mutual learning in PV and clinical trials systems in the four Southern Africa NRAs through collaboration and networking and; 3. To raise the profile of the NRA and PV systems in the four sub-Saharan countries through capacity building of relevant health professionals. SPaRCS used a participatory action learning and co-creation approach. (Vargas et al., 2022). The project was organized into four work packages and each was jointly led by UWC and a partner country colleagues. The main focus was on strengthening system-level capacities so patients and the public were excluded. The project was funded by the European and Developing Countries Clinical Trials Partnership 2 (EDCTP2).

## 3 Project details

**FIGURE 1:**
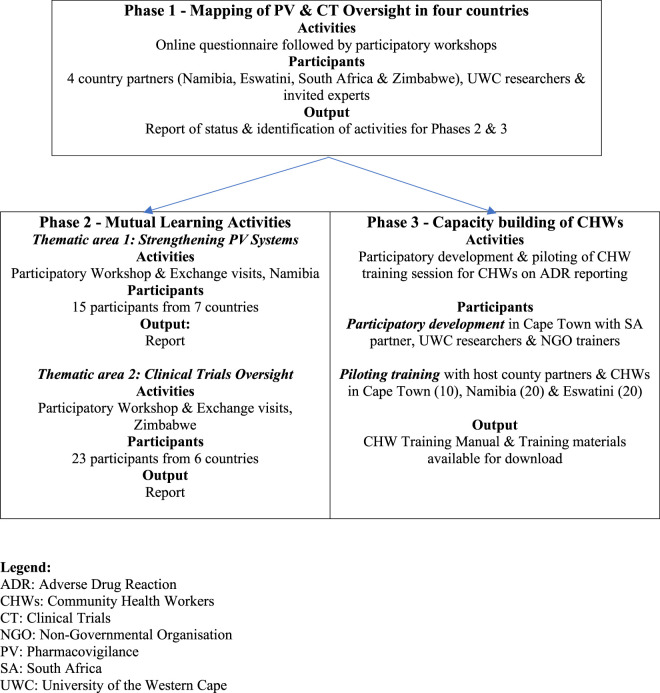
SPaRCS project activities and outputs.

The SPaRCS project comprised three phases (Figure 1).

### 3.1 Phase 1

The objectives of phase one were to map the NRAs for PV and clinical trials oversight in each of the four countries, including structures, personnel, activities and reporting systems, in order to identify areas of good practice, capacity building initiatives and key gaps. The main activities included an online questionnaire followed by a participatory workshop. The tailored online questionnaire, largely based on the WHO PV Indicators Manual, (World Health Organization, 2015), was completed by each of the four SPaRCS partners with input from relevant colleagues in their countries.

The participatory workshop followed the online questionnaire and built on the information that each country had collated. The workshop was originally conceived as a 2-day face to face interaction in which participants would share knowledge and experiences and learn together through interconnected activities. Due to COVID-19 restrictions consequently an alternate virtual participatory workshop program, facilitated via Zoom, had to be implemented. The workshop was divided into four sessions. Sessions 1 and 2 (comprising three activities) took place over 5-h on 19 November 2020 and Sessions 3 and 4 followed in December and January 2021. The workshops were planned and led by UWC and country partners. Each was attended by between 10–14 participants from UWC, the four countries and invited experts from other countries.

**For the first activity,** each country presented a PowerPoint Presentation based on the information gathered from their Data Collection Tool and this was followed by a guided discussion. Key points that emerged were: duplicity of adverse drug reaction (ADR) reporting databases in some countries and a high percentage of ADRs reported by nurses. Commonalities across the four countries included: functional PV centres; involvement in active PV surveillance and PV training but minimal signal detection; lack of PV training in health professional pre-service curricula, and challenges in funding PV activities. Some differences amongst countries were the structure of the NRAs: parastatal versus Ministry of Health (MOH), South Africa had decentralized PV centres, Zimbabwe had considerable expertise in clinical trials, regulatory frameworks and National Ethics Committees (NECs), followed by South Africa. Namibia, on the other hand, actively involved the public in awareness and reporting of ADRs.

**In the second activity,** each country presented the successes and challenges in their PV system and clinical trials oversight and this was followed by reflections. Key successes that emerged: all countries are growing in capabilities in terms of human resources and systems, including use of electronic systems and links to Vigiflow; South African PV and clinical trial systems were considered good but more co-ordination is required, and Namibia’s public awareness initiatives were commendable. Challenges mentioned were almost unanimous and included: financial constraints; lack of capacity building; poor medication error reporting, and poor private sector involvement.

**The third activity** comprised two presentations and a preliminary discussion of potential activities for collaborative learning which was taken forward in subsequent workshops. The first PowerPoint presentation comprised an overview of training options and opportunities in PV and highlighted existing resources in the region. The presentation described two other European and Developing Countries Clinical Trial Partnership (EDCTP) PV projects in the region, PAVIA and PROFORMA (Eswatini is part of both). The importance of following up with like-minded colleagues in the region was discussed as well as ensuring that SPaRCS complements the other EDCTP projects. The second presentation proposed activities suitable for collaborative learning in Phases 2 and 3. After a short discussion the following were identified as potential areas for collaborative learning: increase PV awareness in the four countries; increase involvement of the private sector; funding issues and co-ordination of PV activities, including clinical trials regulation, and ADR monitoring. Two follow-up workshops took place to further distil the topics for collaborative learning in Phase 2 and at this stage it was decided to focus on two fairly broad-based thematic areas: Strengthening PV Systems, and Strengthening Clinical Trials Oversight. These thematic areas were agreed amongst the workshop participants as they provided opportunities for learning and sharing of experiences amongst the four countries.

### 3.2 Phase 2

The focus was on the two selected thematic areas - Strengthening PV Systems, and Strengthening Clinical Trials Oversight. Each thematic area was planned collaboratively by the UWC team and the collaborating country partners.

The *first thematic area* Strengthening PV Systems, commenced with three 2-h workshops, organized virtually (due to COVID-19 travel restrictions) during October and November 2021. The three topics included: PV advocacy; patient reporting of ADRs, and PV data for local regulatory decisions, with speakers from SSA and Europe. These online workshops were attended by approximately 100 participants including project partners and colleagues, invited participants from neighboring countries, and representatives of other relevant EDCTP projects. This was followed-up in July 2023 by an in-person 3-day workshop in Windhoek, Namibia and exchange visits organized by their country partner and colleagues based at the Therapeutic Information and Pharmacovigilance Centre (TIPC), Namibia. Fifteen participants from seven countries (i.e. the four project countries, and Mozambique, Zambia, and Belgium) attended the workshop. Its focus was on sharing country experiences on Strengthening PV Systems, with insightful presentations from the South African Health Products Regulatory Authority (SAPHRA), Autoridade Nacional Reguladora de Medicamento (ANRME, Mozambique) and the Zambia Medicines Regulatory Authority (ZAMRA). The presentations highlighted shared challenges and opportunities across different areas including: the choice and use of reporting systems; use of e-reporting platforms; PV and clinical trials inspections, and communication on safety-related issues, both across stakeholders and to the general public. Overall, the unique value of PV was highlighted, and the exchanges resulted in knowledge sharing and mutual learning. Seven participants stayed on for the subsequent 2-day exchange visits organized by TIPC which included a visit to: an active surveillance support meeting; a marketing authorization holder, and a TB/HIV technical working group.

For the *second thematic area*, Strengthening Clinical Trials Oversight, a 3-day in-person workshop was held in Victoria Falls, Zimbabwe in October 2022. This workshop was planned by the UWC team and the Zimbabwean partners at the Medicines Control Authority of Zimbabwe (MCAZ). MCAZ is designated as a Regional Centre of Regulatory Excellence (RCORE) in Medicine Registration and Evaluation, Quality Assurance/Quality Control and Clinical Trials Oversight under the African Medicines Regulatory Harmonization (AMRH) Initiative of the African Union and the NEPAD Agency. The workshop brought together 23 participants from the NRAs, National Ethics Committees (NECs) and researchers from the four project countries, and aimed to increase collaboration and build on previous efforts to improve the oversight of clinical trials (CTs) in the region. Key learnings from the workshop were an understanding of the status of NRAs and NECs in each of the partner countries. It was clear that all four countries had functioning NRAs supported by legal frameworks and regulations. Although all the countries had functioning PV systems, South Africa appeared to have the most advanced PV system followed by Zimbabwe and Namibia. The NRA in Eswatini had no autonomy as it was a department under the MOH while the other NRAs functioned as semi-independent entities/agencies. Two of the NRAs (South Africa and Zimbabwe) had achieved maturity level 3 (ML3) based on the WHO Global Benchmarking Tool (GBT). Zimbabwe and South Africa had functional clinical trial oversight involving both the NRAs and the NECs. Although Eswatini had legal provisions and regulations for clinical trial oversight, there was limited implementation of procedures for the oversight of clinical trials. In contrast, the NRA in Namibia did not have a legal framework for the regulation of clinical trials. Only the NEC in Namibia has the legal framework for clinical trial oversight. After the workshop, five representatives of the four SPaRCS countries remained in Harare, Zimbabwe for an additional 2-day mutual learning exchange hosted by MCAZ and Medical Research Council of Zimbabwe (MRCZ). Activities included observing a joint inspection of a clinical trial site facilitated by Zimbabwean NEC and NRA colleagues. Finally, the learnings from Phase 2 activities were distilled into key recommendations for SPaRCS partners to share with their country NRAs and PV centres (Table 1).

**TABLE 1:** Key recommendations by SPaRCS to Southern African NRAs and PV centres.

Recommendations by SPaRCS partners to Southern African NRAs and PV centres
Garner greater in-country political support for adequate resources for PV and clinical trials oversight Foster structured collaboration on PV and clinical trials oversight between like-minded stakeholders within countries Invest in south-south collaborations, by drawing in additional regional partners to co-develop or join new initiatives in PV and clinical trials oversight Forge collaborative initiatives through existing African forums such as ZaZiBoNa, SADC and AMRH/AMA. Collaborate to address specific problems related to the adequate use of medicines and vaccines, as a way to prevent adverse reactions Collaborate on communication strategies to health workers and communities regarding controversial/delicate issues that are prone to misinformation (such as safety concerns during the COVID-19 vaccine roll-out)

### 3.3 Phase 3

The focus was on PV capacity building. The SPaRCS partners jointly identified community health workers (CHWs) as the priority target for development and deployment of training materials on ADR reporting. This was partly prompted by the pre-occupation of health professionals with COVID-19 activities during the first 2 years of the project and by a lack of PV training courses and materials specifically targeting CHWs. CHWs are key cadres within SSA health systems and they play a unique and valuable role in directly engaging communities in health matters, as well as in building trust between health systems and local communities. Despite their access to health information at community level, to the best of our knowledge, there are no training materials on PV and ADR reporting targeting CHWs in the region.

The UWC team and the South African partner collaborated with TB HIV Care, a non-profit organization based in Cape Town that employs CHWs to deliver TB and HIV services, (TB HIV Care, 2026), to co-develop a half-day interactive training session on ADR reporting and a complementary Training Manual. The objectives of this interactive training session were: to heighten the awareness of CHWs about adverse drug reactions (ADRs); to know what an ADR is; to equip them with basic knowledge and skills to recognize an ADR, and how and when to report or refer ADRs within their communities and health system. The training session included Power Point presentations, role play and pre- and post-evaluations. The first pilot was carried out in Cape Town with 10 CHWs, and after making adjustments to the training session, a complimentary Training Manual was developed. The complete training package was piloted with two further groups of CHWs, one in Namibia and one in Eswatini (20 participants each), with each pilot coordinated by the respective SPaRCS country partners. These collaborative efforts drew in local NGOs, community-based staff and trainers, who together contributed their valuable *hands-on expertise* to plan and deliver the pilot sessions. The evaluations conducted as part of each pilot training session found that CHWs had gained new knowledge and understanding about PV and ADR reporting and they were enthusiastic to use this in their work settings. Based on these experiences, expanding and embedding training on ADR reporting into CHW activities in other SSA countries is a potential strategy to strengthen PV at regional level, particularly in remote areas. After follow-up discussions amongst the SPaRCS team, the CHW Training Manual and Power Point presentations were finalized and uploaded on the SPaRCS project webpage and are available for download https://soph.uwc.ac.za/project-item/chw-training-material/.

## 4 Discussion

### 4.1 Practical implications

The levels of experience, resource and maturity of NRAs, PV systems and clinical trials oversight varied amongst the four countries. This diversity provided SPaRCS with opportunities for shared learning and collaborative exchanges of challenges, field experience and good practices at sub-national, national and regional level.

#### 4.1.1 Building sustainable relationships amongst partners

Across the different phases and thematic areas, the participatory and co-creation approaches laid the foundation for sharing experiences and learning amongst the diverse group of partners. Moreover, the project allowed us to start, develop and consolidate relationships between partners over a significant period of three and a half years, by working together and meeting in-person, when it became possible after COVID-19. These engagements were particularly welcome, as there are few such opportunities of exchange and harmonization for these professionals, particularly those from small PV units. The project activities, especially the thematic workshops, allowed the project to establish long-term institutional partnerships amongst the four national NRAs/PV centres that go beyond the interest and enthusiasm of their individual representatives. The presence of representatives of African and European academia, not-for-profit organizations and international NGOs at thematic workshops, created additional opportunities for networking with like-minded colleagues. It laid the grounds for establishing an African-rooted, supranational community of practice which can influence PV and regulatory policymakers in the region, much more than isolated efforts.

#### 4.1.2 Sharing of best practices and mutual learning

The SPaRCS consortium was foremost a privileged space of mutual knowledge and exchange. The participants exchanged guidelines in the field of PV, with a focus on the use of electronic platforms, and regulatory oversight, learning from each other’s approaches and processes. They also shared experiences of strategies adopted to stimulate PV reporting and communication. For instance, the experience of the annual #MedSafetyWeek campaign (https://uppsalareports.org/articles/start-small-but-think-big-trials-and-triumphs-of-medsafetyweek-campaigners/) and use of multimedia, e.g. television and radio, to reach out and create awareness among the public on ADR reporting, was shared by Namibia. The use of digital platforms, e.g. cell phone apps and direct web access to facilitate reporting of ADRs, was shared by Zimbabwe and South Africa. Partners from Zimbabwe and Namibia NRAs renewed their commitment to produce quarterly bulletins for healthcare professionals on ADR reporting. This resulted in positive feedback from health professionals and, in Namibia, in a steady increase in ADR reports.

The SPaRCS project was also an incubator for new national and (South-South) international collaboration in clinical trials oversight. For instance, in Zimbabwe, the NEC and NRA conduct joint inspections and audits and during the mutual learning exchange visit to Zimbabwe, the SPaRCS partners observed a joint inspection of a clinical trial site conducted by these respective bodies. As a result, the Eswatini partner indicated that efforts to conduct joint NEC and NRA clinical trial inspections would be explored. However, there was also a realization that joint inspections may not be feasible in all the four countries given the different stages and the structure of the clinical trial oversight framework. For example, the operational structure of the ethics committees in South Africa makes it challenging for the NRA to conduct joint clinical trial inspections with institutional NECs. The ZaZiBoNa experiences in clinical trial oversight were also shared by Zimbabwe and South Africa colleagues. (Dube-Mwedzi and Suleman, 2025).

### 4.2 Lessons learned

A key strength of SPaRCS was the commitment of knowledgeable and highly motivated individuals from the four partner countries who worked together to achieve the project outputs. Moreover, they used their local influence and networks to draw in relevant in-country colleagues as well as reaching out to others in neighboring countries, moving from a national to a regional dimension. This included participation of a representative from Botswana Medicines Regulatory Authority at the Workshop in Zimbabwe; and representatives from Autoridade Nacional Reguladora de Medicamento, Mozambique and Zambia Medicines Regulatory Authority, at the Workshop in Namibia. These participants expressed interest in collaborating in future projects to strengthen partnerships in PV across Southern Africa.

The participatory action learning and co-creation approach promoted a collaborative learning environment which built on each other’s strengths and fostered a community of practice. This approach was based on the understanding that working together for an extended period would strengthen relationships and capacity by learning and reflecting together. This presented a welcome opportunity, in particular, for PV officers from smaller countries, with less mature pharmaceutical systems and fewer resources, who frequently work in isolation.

These collaborative networks demonstrate that it is not necessary (nor feasible) for each country to do all the work alone, or replicate what PV and regulatory centres in neighboring countries have already done. Activities such as: bibliographic searches for evidence supporting a treatment’s safety; identification of publications describing a safety signal under investigation; or developing a communication strategy on critical safety signals, in lay language, for the community, can all be conducted collaboratively. Similarly, brochures or bulletins for communication campaigns can be developed collaboratively at regional level. In addition to minimizing duplication of efforts, this approach will lead to higher quality and more comprehensive and regular outputs, and facilitate a wider reach to more health workers and relevant stakeholders.

A major challenge for SPaRCS was the COVID-19 travel restrictions during the first 2 years. The lack of face-to-face contacts among partners, who were unfamiliar with each other initially, posed a hurdle which was lessened by adapting activities and utilizing online platforms. Online meetings and workshops were more cost effective, had a lower carbon footprint and facilitated engagement with a wider group of participants than SPaRCS had originally envisaged. For example, the online workshops at the beginning of Phase 2 facilitated the participation of PV personnel from West Africa (The Gambia and Liberia) and other SSA countries, including Zambia and Lesotho. The COVID-19 pandemic also prompted the shift to CHWs as the target audience for the capacity building initiative. This shift to a cadre who have historically been overlooked in PV training turned out to be a fortuitous change, as medicine and vaccine safety gained prominence and public awareness during this tumultuous period.

There were several limitations of the SPaRCS project. SPaRCS was a collaboration between NRAs in Namibia, Eswatini and Zimbabwe while in South Africa the main partner was the Pharmacovigilance Centre for Public Health Programs at the National Department of Health and not the NRA, the South African Health Products Regulatory Authority (SAHPRA). During the three and a half years of the project, the main partner from two of the countries (Namibia and Eswatini) resigned and was replaced by a second person. This could have been problematic but with orientation and support from the original partner and the UWC team the new partners fitted into the project. Another limitation was the timing of the study which commenced during COVID-19 so planning and in-person meetings and workshops had to be adjusted to an online platform.

## 5 Conclusions and recommendations

Collaborative work to strengthen knowledge and evidence-based thinking is paramount to enhance the monitoring of the safety and quality of medicines and vaccines, for improving people’s health and for building trust between communities and health systems. This is increasingly important in the era of highly connected and fast-paced societies flooded by information and misinformation about health and health products. The SPaRCS experience indicates that strengthening PV and clinical trials oversight capacity in Southern Africa requires a multifaceted approach involving: the establishment of robust systems; addressing infrastructure and training gaps; integrating PV into public health programs, and learning from successful initiatives from other countries. This regional platform created by SPaRCS provided PV managers based in Southern African regulatory authorities and researchers at academic institutions with the opportunities to identify shared challenges and goals in PV systems and recognize the value of creating a collaborative learning environment. These relationships formed the basis for the establishment of the Centre of Excellence for Pharmacovigilance in Southern Africa (CEPSA), (CEPSA, 2026), located at UWC and led jointly by UWC and the Institute of Tropical Medicine, Antwerp, Belgium, with the aim of strengthening PV activities in the Southern African Region.

## Data Availability

The original contributions presented in the study are included in the article/supplementary material, further inquiries can be directed to the corresponding author.
